# Interactions between Ras and Rap signaling pathways during neurodevelopment in health and disease

**DOI:** 10.3389/fnmol.2024.1352731

**Published:** 2024-02-23

**Authors:** Salvatore J. Cherra, Reagan Lamb

**Affiliations:** Department of Neuroscience, University of Kentucky College of Medicine, Lexington, KY, United States

**Keywords:** guanine nucleotide exchange factor, GTPase activating protein, neurodevelopment, neuropsychiatric disorders, synaptic plasticity, synapse formation

## Abstract

The Ras family of small GTPases coordinates tissue development by modulating cell proliferation, cell-cell adhesion, and cellular morphology. Perturbations of any of these key steps alter nervous system development and are associated with neurological disorders. While the underlying causes are not known, genetic mutations in Ras and Rap GTPase signaling pathways have been identified in numerous neurodevelopmental disorders, including autism spectrum, neurofibromatosis, intellectual disability, epilepsy, and schizophrenia. Despite diverse clinical presentations, intersections between these two signaling pathways may provide a better understanding of how deviations in neurodevelopment give rise to neurological disorders. In this review, we focus on presynaptic and postsynaptic functions of Ras and Rap GTPases. We highlight various roles of these small GTPases during synapse formation and plasticity. Based on genomic analyses, we discuss how disease-related mutations in Ras and Rap signaling proteins may underlie human disorders. Finally, we discuss how recent observations have identified molecular interactions between these pathways and how these findings may provide insights into the mechanisms that underlie neurodevelopmental disorders.

## Introduction

The Ras superfamily of G proteins was named for its founding members: HRas, NRas, and KRas. This family of proteins was originally discovered as viral and human oncogenes (Harvey, 1964; Kirsten and Mayer, 1967; Scolnick et al., 1973; Der et al., 1982; Parada et al., 1982; Santos et al., 1982). Besides their early roles in cell proliferation, Ras proteins also play roles in many signal transduction pathways to regulate differentiation, cell migration, and intracellular trafficking. A close family member, Rap, was initially identified as a Ras antagonist (Kitayama et al., 1989). Since their discovery, Rap G proteins were shown to regulate cell proliferation, adhesion, and polarity. In the nervous system, Ras and Rap modulate the proliferation of neural progenitors, development of synapses, and formation or maintenance of plasticity through distinct signaling pathways. Mutations in Ras or Rap and associated regulatory proteins were identified in multiple neurological disorders (Table 1).

**Table 1:** A summary of Ras and Rap related neurological disorders.

**Gene**	**Disorders implicated**	**Ras or Rap**	**References**
Neurofibromin	Neurofibromatosis-1	↑Ras	Martin et al., 1990
			Xu et al., 1990
			Diggs-Andrews and Gutmann, 2013
			Diggs-Andrews et al., 2014
KRas/NRas	Noonan syndrome	↑Ras	Cirstea et al., 2010
			Lee B. H. et al., 2011
			Wingbermuhle et al., 2022
PTPN11	Noonan syndrome	↑Ras	Pierpont et al., 2009
	Intellectual disability		Lee B. H. et al., 2011
	Developmental delay		Rauen, 2013
			Lee et al., 2014
			Altmuller et al., 2017
			Wingbermuhle et al., 2022
SOS1	Noonan syndrome	↑Ras	Roberts et al., 2007
			Tartaglia et al., 2007
			Zenker et al., 2007
			Lee B. H. et al., 2011
			Wingbermuhle et al., 2022
RAF1	Noonan syndrome	↑Signaling downstream of Ras	Pandit et al., 2007
			Lee B. H. et al., 2011
			Holter et al., 2019
			Guo et al., 2021
			Wingbermuhle et al., 2022
SPAR	Anxiety	↑Ras	Matsuura et al., 2022
SynGAP	Intellectual disability	↑Ras & ↑Rap	Komiyama et al., 2002
	Autism spectrum		Hamdan et al., 2009
	Epilepsy		Hamdan et al., 2011
	Schizophrenia		de Ligt et al., 2012
			Carvill et al., 2013
			Berryer et al., 2013
			Ozkan et al., 2014
			Jeyabalan and Clement, 2016
			Kilinc et al., 2018
			Brimble et al., 2019
			Araki et al., 2023
Epac1/RAPGEF3	Depression	↓Rap	Middeldorp et al., 2010
	Anxiety		
Epac2/RAPGEF4	Autism spectrum	↓Rap	Bacchelli et al., 2003
	Depression		Dwivedi et al., 2006
	Anxiety		
Rap1	Depression	↓Rap	Dwivedi et al., 2006
RAPGEF2	Schizophrenia	↓Rap	Xu et al., 2008
			Xu et al., 2009
RAPGEF6	Schizophrenia	↓Rap	Xu et al., 2008
			Xu et al., 2009

The Ras superfamily influences many signaling pathways based on its members' interactions with downstream effector proteins, like protein or phospholipid kinases. While protein interaction domains mediate the interactions between small G proteins and their effectors, the availability of these interaction domains on G proteins is locally and temporally regulated. Binding of GTP to small G proteins causes a conformational change that exposes these binding domains and enables G proteins to dock with their effector proteins (Geyer et al., 1996; Spoerner et al., 2007). Hydrolysis of GTP to GDP changes the G protein structure into an inactive state. The binding and hydrolysis of GTP is modulated by guanine nucleotide exchange factors (GEFs) and GTPase activating proteins (GAPs), respectively [for more details see Rhett et al. (2020)]. To fully understand how G proteins, including Ras family members, mediate the effects of various signal transduction pathways, one must consider the roles of proteins involved: the GTPases, the effectors, and the GEFs and GAPs that modulate the GTPases. Here, we present specific Ras- and Rap-associated neurological disorders and highlight gaps in our knowledge within and between the proposed signaling pathways for these small G proteins. We also discuss how crosstalk or co-regulation of Ras and Rap functions coordinate neurodevelopment and how imbalances between Ras and Rap signaling may provide a new framework for understanding certain neurodevelopmental disorders.

## Ras signaling during development and plasticity

**Figure 1:**
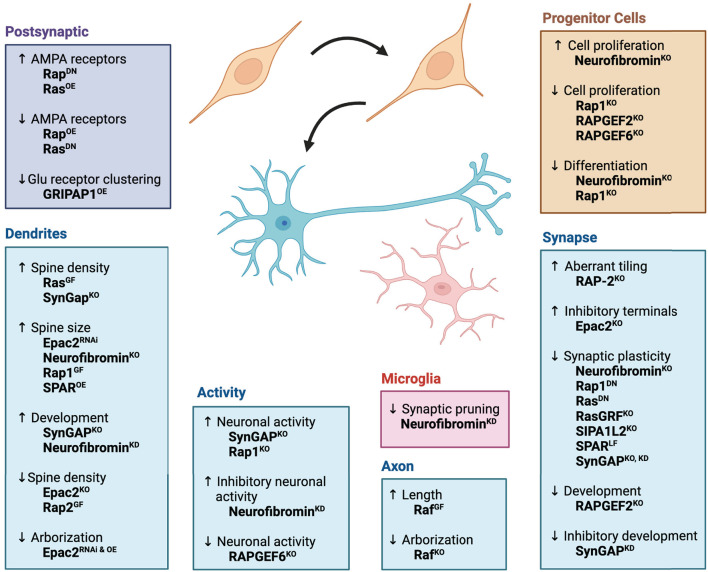
Effects of Ras/Rap signaling pathways on neurodevelopment. Ras, Rap, and their associated signaling pathways promote nervous system function by regulating progenitor cell proliferation, neuronal differentiation, synapse formation, and synaptic plasticity. The diagram illustrates roles for each protein at different developmental steps or in different cellular compartments of the neuron. Superscripts denote genetic approaches used to study protein function: OE, overexpression of gene; DN, expression of dominant negative gene; KO, genetic knockout; GF, expression of gain-of-function mutation; LF, expression of loss-of-function mutation; RNAi, RNAi-mediated knockdown of gene expression. Figure was created using BioRender.

Ras signaling coordinates the survival, growth, and development of many tissues, including neurons (Zhong, 2016). Early studies found that mice lacking a Ras GEF, Ras-GRF (guanine nucleotide releasing factor), showed impaired synaptic plasticity, implying that Ras activation is necessary for synaptic plasticity (Brambilla et al., 1997). Subsequent research showed that Ras activation in neurons leads to hypertrophy of the neurons and their neurites through a MAPK signaling pathway (Heumann et al., 2000; Gartner et al., 2004). Activation of Ras also increased synapse formation, dendritic spine density, neurotransmission, and drove synaptic plasticity (Zhu et al., 2002; Arendt et al., 2004; Seeger et al., 2004, 2005; Harvey et al., 2008). The downstream target of Ras, Raf, functions to promote axon elongation and arborization (Zhong et al., 2007; O'Donovan et al., 2014). Proper synaptic development and plasticity also require GEFs and GAPs that modulate Ras activity. For example, neurons from SynGAP knockout mice, which lack this negative regulator of Ras, produced more dendritic spines and displayed higher neuronal activity (Vazquez et al., 2004). However, another study found reduced synaptic plasticity in neurons from a different SynGAP knockout mouse (Kim et al., 2003). Altogether, these and many other studies highlight the importance of Ras-Raf-MAPK signaling as an essential component for neuronal development and function (Figure 1).

### Ras GAP function in neurofibromatosis

Neurofibromatosis-1 is a genetically heritable disorder associated with mutations in neurofibromin, a Ras GAP-containing protein (Ballester et al., 1990; Martin et al., 1990; Xu et al., 1990). Neurofibromatosis patients display a predisposition to cancer and impairments in attention, learning, and memory (Diggs-Andrews and Gutmann, 2013). Deletion of one copy of neurofibromin in mice produces social learning deficits and metabolic alterations observed in human patients (Molosh et al., 2014; Tritz et al., 2021). Although neurofibromatosis-1 does not display a sex bias in frequency of occurrence, male humans and mice exhibit a higher propensity for cognitive impairments (Diggs-Andrews et al., 2014). More recently, sex differences in neurofibromin heterozygote mice were shown to parallel observations in human patients, where males tend to display cognitive impairments, like memory deficits, females display anxiety or anxiety-like behaviors (Santos et al., 2023). While more information on sex differences is needed, these studies indicate that phenotypic impairments caused by neurofibromin mutations are highly conserved.

At the cellular level, these deficiencies can be attributed changes in neuroglial progenitor cell proliferation and differentiation (Bennett et al., 2003; Hegedus et al., 2007; Lee et al., 2010). Ras signaling through ERK appears to mediate the regulation of progenitor cells (Hegedus et al., 2007; Lee et al., 2010). In neurofibromin knockout mice, perturbations in a Ras-MAPK signaling pathway underlie deficits in neurotransmission, synaptic plasticity, or impairments in dendritic spine enlargement (Costa et al., 2002; Guilding et al., 2007; Cui et al., 2008; Oliveira and Yasuda, 2014). Additional studies suggest AKT, Pak1, cAMP/PKA, and other pathways also may play roles downstream of neurofibromin (Guo et al., 1997; The et al., 1997; Tong et al., 2002; Hegedus et al., 2007; Brown et al., 2010, 2012; Wang et al., 2012; Molosh et al., 2014). Bergoug et al. (2020) provided a detailed view of the structural and functional aspects of neurofibromin. In more recent work, neurofibromin may act independent of the Ras signaling pathway to regulate protein synthesis in conjunction with the valosin-containing protein to promote spine formation and social memory (Shih et al., 2020). Neurofibromin haploinsufficiency also was found to disrupt a critical development period in cortical neurons by upsetting excitation-inhibition balance due to increased activity of inhibitory neurons (Van Lier et al., 2020).

In addition to neurons, neurofibromin mutations also impact microglial function. For example, microglial phagocytic activity, which is associated with synaptic pruning, was reduced in neurofibromin heterozygote mutants (Elmadany et al., 2020). Using microglia derived from human induced pluripotent stem cells, Kuhrt et al. (2023) showed that microglia derived from human neurofibromatosis patients also displayed reduced phagocytic activity. Beyond the occurrence of gliomas, these studies suggest that neurofibromin mutations in glia may contribute to neurological deficits.

Focusing on Ras activity as the potential tipping point for neurofibromatosis caused by mutations in neurofibromin, one unanswered question is whether the function of a Ras GEF is required for elevated Ras signaling in the neurofibromin mutant background. A Ras GEF called GRIPAP1 (glutamate receptor interacting protein 1 associated protein) modulates AMPA receptor localization in mouse neurons (Ye et al., 2000). Interestingly, human GRIPAP1 is located on the X-chromosome, suggesting a possible connection between the impairments of neuronal function and biological sex in neurofibromatosis patients. While this question of Ras GEF involvement has recently been posed in the context of neurofibromatosis-related cancer (Jackson et al., 2023), its application to neurodevelopment and plasticity is not known.

### Ras signaling in Noonan syndrome

Noonan syndrome is a heritable disorder that primarily affects tissues outside of the nervous system, but some cases display cognitive impairment (Rauen, 2013). More than half of the cases are associated with mutations in a Ras signaling pathway that includes PTPN11 (protein tyrosine phosphatase non-receptor type 11), SOS1 (son of sevenless homolog 1), RAF1, KRAS, NRAS, SHOC2 (suppressor of clear homolog 2), and CBL (casitas B-lineage lymphoma proto-oncogene) [for more details see additional reviews Rauen (2013) and Wingbermuhle et al. (2022)]. Gain-of-function mutations in KRAS(T58I), NRAS(T50I or G60E), or RAF1(P261S or L613V) directly lead to increased MAPK signaling (Pandit et al., 2007; Cirstea et al., 2010). SOS1 is a Ras GEF, which is overactivated by mutations associated with Noonan syndrome (Roberts et al., 2007; Tartaglia et al., 2007; Zenker et al., 2007). These gain-of-function mutations subsequently increase Ras signaling through the Ras-Raf-MAPK pathway.

In mice, expression of a KRAS(V14I), SOS1(E846K), or RIT1(A57G) mutation produces many Noonan syndrome-related phenotypes, like growth delay, craniofacial abnormalities, and cardiac defects (Chen et al., 2010; Hernandez-Porras et al., 2014; Takahara et al., 2019). Expression of PTPN11 activating mutations cause varying levels of in the memory impairment depending on targeted brain region (Lee et al., 2014; Altmuller et al., 2017). However, when investigating Noonan syndrome-associated Raf mutations, Holter et al. (2019) observed enhanced spatial and working memory and fear learning in Raf1(L613V) heterozygote mice, suggesting enhanced cognitive functions rather than the cognitive impairments observed in most RAF1-associated Noonan syndrome patients. While the phenotypes of many mutant mouse models correlate with human patients, the discrepancies with Raf1, SOS1, or other genes suggest more information is needed to understand how these genes influence Noonan syndrome phenotypes and how the pathway as a whole regulates nervous system function. The development of new Noonan syndrome-related resources, like human induced pluripotent stem cells, may provide additional insights into the molecular mechanisms in human tissues (Guo et al., 2021; Kim et al., 2022; Busley and Cyganek, 2023; Sbrini et al., 2023).

While mutations in the Ras-MAPK signaling pathway underlie many Noonan syndrome phenotypes, not all Noonan-associated mutations in this pathway cause neurological deficits (Wingbermuhle et al., 2022). For example, patients with SOS1 mutations achieved near normal intellectual scores, but mutations in PTPN11 were associated with lower intellectual scores (Pierpont et al., 2009). An additional study investigating more genes in the Ras-MAPK pathway found that mutations in all genes investigated, except SOS1, are associated with developmental delay or intellectual disability (Lee B. H. et al., 2011). This exception may hint at unanswered questions regarding developmental stage-specific roles (Tian et al., 2004) or even neuron-specific roles for Ras GEFs, like Sos1/2 or Ras-GRF1/2. While activating mutations for the Ras-MAPK pathway have been identified through the genetics of Noonan syndrome, it's not clear what roles Ras GAPs may play. For example, p120-Ras-GAP/RASA1 function/localization was proposed to be altered as a cause for growth delay in Noonan PTPN11 mutant mice (De Rocca Serra-Nedelec et al., 2012), but its effects on intellectual abilities still have not been tested.

### Ras GAP function in SynGAP-related neurological disorders

Mutations in SynGAP are associated with multiple neurodevelopmental disorders, like intellectual disability, autism spectrum, epilepsy, and schizophrenia (Jeyabalan and Clement, 2016; Kilinc et al., 2018). Many of these mutations result in premature stop codons that lead to reductions in mRNA or protein expression. For example, exon mutations like K138X, R579X, L813RfsX23, Q893Rfs, K108Vfs, W267X, or R143X directly lead to truncated proteins (Hamdan et al., 2009, 2011; Carvill et al., 2013). Other mutations found in SynGAP-related disorders disrupt proper mRNA splicing, like c.501–1G-A or 2294 + 1G-A, which likely lead to exon skipping and/or incorporation of frameshifts that lead to premature terminations (Hamdan et al., 2011; de Ligt et al., 2012). Interestingly, Berryer et al. (2013) found two disorder-associated non-synonymous mutations, W362R and P562L, which affect the C2 domain and the Ras/Rap binding groove. However, no expression of these mutant proteins was detected when they were introduced to cultured neurons, suggesting these mutant proteins were unstable. Since most clinical SynGAP mutations are presumed to reduce protein expression, many studies have turned to SynGAP heterozygote knockout mice to model clinical haploinsufficiency. In mice, SynGAP haploinsufficiency results in phenotypes consistent with cognitive impairment and epilepsy, suggesting that reduced expression or function of SynGAP1 underlies these disease-associated phenotypes (Clement et al., 2012; Ozkan et al., 2014; Aceti et al., 2015). Araki et al. (2023) directly tested the effects of SynGAP1 mutations identified in patients with intellectual disabilities: L813RfsX22 and c.3583–9G>A, which both induce premature stop codons (Hamdan et al., 2009; Brimble et al., 2019). Like the heterozygote knockout mice, these disease-associated knock-in mice also display hyperactivity and impairments in working memory (Araki et al., 2023).

SynGAP was identified as a Ras GAP that localizes to synapses (Chen et al., 1998; Kim et al., 1998) [for more information see a detailed review (Gamache et al., 2020)]. Reductions in synaptic plasticity observed in SynGAP haploinsufficiency mice coincide with decreases in Ras activity and downstream MAPK activation (Komiyama et al., 2002; Ozkan et al., 2014). However, SynGAP also was shown to act on both Ras and Rap with the ability to differentially modulate each G protein based on posttranslational modifications (Krapivinsky et al., 2004; Walkup et al., 2015). At the cellular level, SynGAP haploinsufficiency in mice alters dendritic spine dynamics and maturation (Clement et al., 2012). While earlier studies focused on deficiencies in excitatory synapses (Kim et al., 1998; Vazquez et al., 2004; Rumbaugh et al., 2006), recent work has found that SynGAP also plays a role in the formation and function of inhibitory synapses (Berryer et al., 2016; Khlaifia et al., 2023). Therefore, more information is needed to fully understand how SynGAP function in specific populations of neurons influences the formation and function of circuits associated with intellectual disabilities and other SynGAP-related disorders. Further examination of the signaling pathways that interact with SynGAP, like Ras or Rap, their respective GEFs, and downstream effectors, is needed to understand how this network of proteins promotes neuronal function and plasticity at the molecular level.

## Rap signaling during development and plasticity

An initial study found that Rap1-MAPK signaling is essential for neuronal differentiation (York et al., 1998). More recently Rap1 was shown to regulate synaptic function and plasticity (Morozov et al., 2003; Subramanian et al., 2013). While Rap1 activation showed no effects on dendritic arbors, a dominant negative mutant increased the overall length of dendrites (Fu et al., 2007). Interestingly, increases and decreases in Rap2 activity influenced dendritic arbors in which activation of Rap2 decreased dendrite length and a Rap2 dominant negative mutant increased dendrite length (Fu et al., 2007). In *C. elegans*, RAP-2 was found to restrain the placement or tiling of synapses to limit the overlap of synapses between neighboring neurons (Chen et al., 2018). Further support for Rap functions at the synapse come from studies on Rap GAPs. The Rap-specific GAP, SPAR [Signal-induced proliferation-associated 1(SIPA1)-like 1], localizes to dendritic spines where it caused enlargement of the spines (Pak et al., 2001; Pak and Sheng, 2003; Maruoka et al., 2005). SPAR also acts as an integration point for other signaling pathways that regulate synaptic plasticity (Seeburg et al., 2008). Another Rap GAP, SIPA1L2 (SIPA1-like 2), was found to promote synaptic plasticity by regulating intracellular trafficking of membranous organelles (Andres-Alonso et al., 2019). Highlighting the need for bidirectional regulation of GTPases, Mayanagi et al. (2015) found that RAPGEF2 and SPAR interact with the same postsynaptic density protein to control the activity of Rap2 during spine development. Homologs of RAPGEF2 were also shown to promote presynaptic development in *Drosophila* and *C. elegans* (Heo et al., 2017; Ou et al., 2019; Lamb et al., 2022). Additional Rap GEFs promote dendrite development and synaptic transmission (Woolfrey et al., 2009; Srivastava et al., 2012b). Altogether, modulation of Rap signaling is essential for proper neuronal development and function (Figure 1).

### Rap GEF function in autism spectrum disorder

The family of Epac (exchange protein activated by cAMP) proteins, which includes Epac1 and Epac2, was originally identified as cAMP-activated GEFs for Rap GTPases (de Rooij et al., 1998, 2000; Kawasaki et al., 1998). More recently, four rare mutations in Epac2/RapGEF4 were identified that associated with autism spectrum phenotypes (Bacchelli et al., 2003). Heterozygote and homozygote Epac2 knockout mice display impairments in social behaviors (Srivastava et al., 2012a). However, using a different Epac2 knockout mouse, another study found that social deficits are only present when Epac1/RapGEF3 and Epac2 are deleted, suggesting potential functional redundancy (Yang et al., 2012).

Knockdown of Epac2 or expression of one of the autism-associated Epac2 mutations reduced dendritic arborization, increased the size of dendritic spines, and reduced AMPA receptor-mediated excitatory neurotransmission (Woolfrey et al., 2009; Srivastava et al., 2012b). More recently, Jones et al. (2019) observed an increase in the number of inhibitory presynaptic terminals labeled by the vesicular GABA transporter, but no changes in excitatory presynaptic terminals labeled by the vesicular glutamate transporter in Epac2 knockout neuronal cultures. The decreased ratio between excitatory and inhibitory synapses agrees with previous studies on excitation-inhibition balance and social impairments associated with autism spectrum (Yizhar et al., 2011). While the disease association and behavioral results from mutant mice require further investigation, the results from cellular and molecular studies support its roles in autism-related cellular phenotypes.

### Rap1 and Rap GEFs in anxiety and depression

Reduced expression of Rap1 and Epac2 have been associated with depression in suicide victims (Dwivedi et al., 2006). Another study using genome wide association analysis identified single nucleotide polymorphisms in Epac1 that associated with depression and anxiety (Middeldorp et al., 2010). However, between the two populations tested, the associations between single nucleotide polymorphism and phenotype lacked agreement or were linked to different effects in each population. In support of the human studies, Epac2 knockout mice display anxiety- and depression-like behaviors (Zhou et al., 2016). Interestingly, mice lacking the Rap GAP, SPAR1, display multiple behavioral deficits, including increased anxiety (Matsuura et al., 2022). Together these studies suggest that dysregulation of Rap signaling may precipitate anxiety or depression behaviors. However, further studies will be required to determine if alterations in Rap1 or Rap2 signaling underlie anxiety or depression, how additional Rap GEFS or GAPs influence the development of these phenotypes, and which downstream effectors may be involved.

### RapGEFs in schizophrenia

Copy number variations in RAPGEF2 and RAPGEF6, which belong to a well conserved PDZ-RAPGEF family, are associated with schizophrenia (Xu et al., 2008, 2009). Conditional knockout of either RAPGEF2 or RAPGEF6 in mice caused hyperlocomotion and deficits in learning and memory, which have been likened to symptoms observed in schizophrenia patients (Maeta et al., 2018). At the cellular level, deletion of RAPGEF6 in mice displayed impaired neuronal activity in the hippocampus and amygdala, which are implicated in schizophrenia (Levy et al., 2015). Mutations in *Drosophila* and *C. elegans* RAPGEF6 homologs disrupt synaptic development and reduce neuronal activity (Heo et al., 2017; Ou et al., 2019; Lamb et al., 2022). Additionally, RAPGEF2, RAPGEF6, and Rap1 are essential for the development of neural progenitor cells, which has recently been hypothesized as a risk factor for schizophrenia (Maeta et al., 2016; Shah et al., 2017; Dietz et al., 2020). Together, these studies highlight possible mechanisms through which impairments in Rap or its GEFs may lead to neurological disorders; however, the roles of Rap GAPs and downstream effectors still require further investigation.

## Convergence of Ras and Rap signaling pathways

Almost 20 years after the discovery of Ras, Rap was identified as an antagonist for the oncogenic effects of Ras (Kitayama et al., 1989). Both Ras and Rap play important roles in neurodevelopment and synaptic plasticity, but it wasn't clear if this antagonism held true for the nervous system. Zhu et al. (2002) found that Ras and Rap indeed play antagonizing roles for synaptic plasticity, where Ras mediates the delivery of AMPA receptors to postsynaptic sites and Rap mediates the removal of AMPA receptors. Therefore, balance of Ras and Rap signaling events appears to be critical for the regulation of synaptic function. However, it wasn't until more recently that studies began identifying molecular mechanisms that co-regulate Ras and Rap. For example, phosphorylation by polo-like kinase 2 was identified as one possible mechanism to modulate the balance of Ras and Rap signaling. Phosphorylation of the Ras GEF, RasGRF1, and the Rap GAP, SPAR, by polo-like kinase 2 led to the degradation of RasGRF1 and SPAR (Lee K. J. et al., 2011). Reductions in Ras GEF activity would lead to a decrease in Ras signaling, and reductions in Rap GAP activity would increase in Rap signaling. These findings suggest that individual signaling proteins, like kinases, can tip the balance between Ras and Rap signaling as required for the development of the nervous system. Interestingly, dysregulation of Ras and Rap signaling disrupts synaptic plasticity and impairs memory.

Co-regulation of Ras and Rap can occur at the level of individual, bifunctional GTPase modulators. While some GAPs are specific for Ras or Rap, others display GAP activity toward both G proteins. For example, SynGAP can act as a GAP for either Ras or Rap (Chen et al., 1998; Kim et al., 1998); however, it wasn't clear if SynGAP always acted on both GTPases or if its GAP activity could be tuned for each target. More recently, phosphorylation of SynGAP was shown to alter its GAP activity toward Ras and Rap in a kinase-dependent manner (Walkup et al., 2015). CDK5 phosphorylation of SynGAP favored inactivation of Ras more than Rap, but CaM kinase II phosphorylation of SynGAP resulted in more Rap inactivation than Ras inactivation (Walkup et al., 2015). Together, these two kinases, and possibly others, may co-regulate Ras and Rap signaling pathways by tuning the specificity of bifunctional GAPs.

In addition to SynGAP, most members of the GAP1/RASA family, including RASA3, CAPRI/RASA4, and RASAL display bifunctional GAP activity toward Ras and Rap (Kupzig et al., 2006). Interestingly, the specificity of GAP activity of CAPRI/RASA4 is dependent on calcium (Dai et al., 2011). Monomeric RASA4 acts predominantly as a Ras GAP, but calcium induces dimerization of RASA4 and increases its activity toward Rap (Dai et al., 2011). While these effects have not been investigated in the nervous system, the calcium-dependent switch in GAP activity provides an intriguing mechanism to modulate the balance of Ras and Rap activities. Together with SynGAP, this study suggests that modulation of bifunctional GAPs through post-translational modifications or second messenger systems may provide cells with multiple mechanisms to co-regulate Ras and Rap activities.

Other possible mechanisms that link Ras and Rap functions come from protein interaction studies. For example, Ras directly interacts with Epac, which modulates Rap function, to regulate dendritic arbors (Srivastava et al., 2012b). More recently, SHANK3 (SH3 and multiple ankyrin repeat domains 3) was found to bind Ras and Rap (Lilja et al., 2017). While no differential regulation was described, this binding could provide an additional mechanism to sequester active Ras and Rap proteins from specific cellular locations. Finally, Zhang and colleagues reported that Ras and Rap promote synaptic plasticity through their functions in different microdomains (Zhang et al., 2018). Together, these studies suggest that protein-protein interactions or sequestration of Ras, Rap, and associated proteins may provide additional mechanisms that modulate Ras and Rap activities and provide future directions to determine how the balance between Ras and Rap signaling coordinates the development of the nervous system. Overall, these studies represent a shift in our understanding from insular Ras and Rap signaling pathways of antagonism toward an interconnected signaling network whose balance dictates essential developmental processes in the nervous system.

## Discussion

Based on genome-wide association studies, modulators and effectors of Ras and Rap signaling pathways have been implicated in multiple neurological disorders. Together with fundamental studies on Ras and Rap, these disease-association studies highlight the importance of these pathways in neurodevelopment and synaptic function. The development of new genetic models to study disorders associated with Ras and Rap dysregulation present new ways to understand this complex signaling network, identify therapeutic targets for historically “undruggable” genes, and resolve many of the currently unanswered questions. For example, Rap has been shown to antagonize the effects of Ras signaling. Does an imbalance between these two GTPases underlie neurological disorders associated with Ras hyperactivity? Could restoring this balance correct intellectual disabilities or impairments in learning and memory? On the other hand, hypoactivity of Rap appears to be the consensus for many Rap-associated disorders. Is this simply a variation on the same theme of balance between Ras and Rap? Or do the underlying causes stem from the impairment of distinct Rap signaling pathways? While the complexity of these pathways is continuously being redefined, their intersections provide new views into the fundamental aspects of neurodevelopment. Finally, answering these basic questions about how small GTPases are modulated, how they coordinate essential developmental processes, and how they interact with each other should uncover new insights for approaching neurological disorders.

## Author contributions

SC: Conceptualization, Funding acquisition, Writing—original draft, Writing—review & editing. RL: Conceptualization, Funding acquisition, Writing—original draft, Writing—review & editing.
